# Bacterial Cellulose Production from agricultural Residues by two *Komagataeibacter* sp. Strains

**DOI:** 10.1080/21655979.2022.2062970

**Published:** 2022-04-13

**Authors:** Moyinoluwa O. Akintunde, Bukola C. Adebayo-Tayo, Mofoluwake M. Ishola, Akram Zamani, Ilona Sárvári Horváth

**Affiliations:** a Department of Microbiology, University of Ibadan, Ibadan, Nigeria; b Swedish Centre for Resource Recovery, University of Borås, Borås, Sweden; c Department of Energy and Environment, Göteborg Energi, Gothenburg, Sweden

**Keywords:** Agricultural residue, bacterial cellulose, *komagataeibacter*, agitation, corn cob

## Abstract

Agricultural residues are constantly increasing with increased farming processes, and improper disposal is detrimental to the environment. Majority of these waste residues are rich in lignocellulose, which makes them suitable substrate for bacterial fermentation in the production of value-added products. In this study, bacterial cellulose (BC), a purer and better form of cellulose, was produced by two *Komagataeibacter* sp. isolated from rotten banana and kombucha drink using corncob (CC) and sugarcane bagasse (SCB) enzymatic hydrolyzate, under different fermentation conditions, that is, static, continuous, and intermittent agitation. The physicochemical and mechanical properties of the BC films were then investigated by Fourier Transformed Infrared Spectroscopy (FTIR), Thermogravimetry analysis, Field Emission Scanning Electron Microscopy (FE-SEM), and Dynamic mechanical analysis. Agitation gave a higher BC yield, with *Komagataeibacter* sp. CCUG73629 producing BC from CC with a dry weight of 1.6 g/L and 1.4 g/L under continuous and intermittent agitation, respectively, compared with that of 0.9 g/L in HS medium. While BC yield of dry weight up to 1.2 g/L was obtained from SCB by *Komagataeibacter* sp. CCUG73630 under continuous agitation compared to that of 0.3 g/L in HS medium. FTIR analysis showed BC bands associated with cellulose I, with high thermal stability. The FE-SEM analysis showed that BC fibers were highly ordered and densely packed. Although the BC produced by both strains showed similar physicochemical and morphological properties, the BC produced by the *Komagataeibacter* sp. CCUG73630 in CC under intermittent agitation had the best modulus of elasticity, 10.8 GPa and tensile strength, 70.9 MPa.

## GRAPHICAL ABSTRCAT

**Figure:**
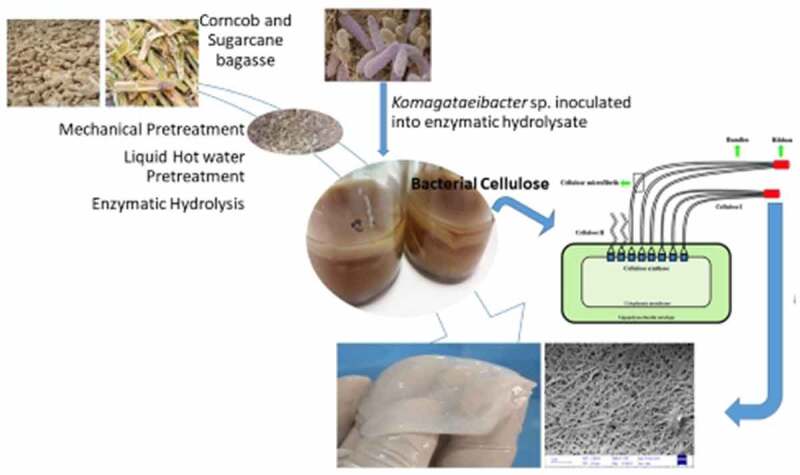

## 1. Introduction

Cellulose is a major component of plant cell wall and hence the most abundant polymer on earth, about 1.5 trillion tons of cellulose is produced annually [1,2]. Cellulose can also be synthesized by some fungi, bacteria, tunicates, and algae. Bacterial cellulose (BC) is the most common among the non-plant sources of cellulose, it serves as a suitable alternative for plant cellulose in various pharmaceutical and industrial applications. Moreover, BC is purer and has better physicochemical properties like higher crystallinity, tensile strength, and water-holding capacity over plant cellulose, although both these types present similar structural properties as they are made up of glucose monomers linked together by β-1, 4 glycosidic linkages [3].

Several bacteria of different genus can produce cellulose [4–6], but only strains of the genus *Gluconacetobacter*, with some strains renamed as *Komagataeibacter*, [7] have been found to produce promising quantities of cellulose [8,9]. Regarding these promising strains, investigations for the utilization of cost-effective feedstock, as an alternative to expensive synthetic media, is very important. Agricultural residues are rich in lignocellulosic materials and are suitable substrates in different biological processes. Renewable low-cost agricultural residues and industrial by-products, such as fruit peels and juice, rice husk, molasses, wheat straw, palm date fruits, olive oil mill wastewater have been studied previously as substrates for BC production [10–14]. Furthermore, there is an increased interest in the commercial applications of BC; thus, new cost-effective production technologies using innovative and cheap feedstocks, as well as the scaling up of bioprocess techniques for industrial applications have become more and more important [15].

About 2 billion tons of agricultural wastes are accumulated globally, with an increase in the numbers over time [16]. With intensified farming to combat hunger, there is a continuous development in crop production. This will in turn result in high amounts of agricultural wastes with negative environmental effect if not properly managed. Therefore, there is a need for these wastes to be transformed into sustainable value-added products. Nigeria is the largest producer of corn in Africa, with a production of 10.5 million metric tons in 2017 [17]. The cob is the waste generated from corn processing after removing the grains. Furthermore, Nigeria is the second-largest sugar market in sub-Saharan African after South Africa, with sugarcane is mostly grown in Northern Nigeria where the weather and soil condition are most conducive. Foreign Agricultural Service (FAS) Lagos forecasts Nigeria’s domestic cane sugar production in marketing year (MY) 2021/22 to reach roughly 70,000 metric tons (raw value), down about 7% compared to 75,000 metric tons in MY 2020/21 [18]. However, increasing sugar production through sugarcane farming, will lead to generation of large amounts of bagasse waste, as 1 ton of sugarcane will generate 270 kilos of bagasse [19].

Corncob (CC) and sugarcane bagasse (SCB) are agro-wastes rich in lignocellulosic residues, generated in large quantities. In Nigeria, they are usually left to dry on the farm before been burnt off or they are found littering the streets and drainages [20,20]. This ineffective disposal methods results in environmental pollution, which further contributes to global climate challenges.

The challenge in utilization of lignocellulosic residues is their recalcitrant structure. These types of wastes must therefore undergo a pre-treatment, to break down their complex structure [21]. Lignocellulosic materials are composed of cellulose, hemicellulose, and lignin, which are associated with each other in a heteromatrix. The aim with introducing a pre-treatment process step is to increase the accessibility of cellulose and hemicellulose for efficient enzymatic hydrolysis, as hydrolysis releases fermentable sugars, which in turn can be utilized by the microorganisms [22].

Even though lignocellulosic materials have been used for BC production, in most of these cases, non-sustainable hydrolysis methods were applied [23,24]. 24,used CC acid hydrolyzate for BC production, 25,also used acetic acid pretreated bagasse for BC production, in previous studies. However, these acid hydrolyzates cannot be used directly for BC production, hence they need to be detoxified prior to their utilization. This additional process step would negatively affect the BC production process both environmentally and economically, there is therefore a need to investigate other chemical-free approaches within the BC production process.

There has been no study on enzymatic hydrolysis combined with mechanical pre-treatment of CC and SCB for production of BC. The aim of this study was therefore to investigate the effects of different pre-treatment steps using environmentally friendly methods prior to enzymatic hydrolysis. The hydrolyzates obtained from CC and SCB were then used as substrates for the bacterial growth and cellulose production under three different fermentation conditions that is, static or continuous, as well as intermittent-agitations, in order to optimize BC production by *Komagataeibacter* sp. Moreover, since the effect of different fermentation conditions on the physicochemical properties of BC has not yet been investigated, BC was finally characterized using Fourier Transformed Infrared Spectroscopy (FTIR), Field-Emission Scanning Electron Microscopy (FE-SEM), and Thermogravimetric Analysis (TGA). Furthermore, the mechanical properties which were determined with a Dynamic Mechanical Analyzer (DMA).

### 1.1 List of abbreviations

BC – Bacterial Cellulose

CC – Corncob

SCB – Sugarcane bagasse

LHW – Liquid Hot Water

MWA – Microwave Assisted

## 2. Materials and methods

### 2.1 Materials

The CC used was obtained from dumpsite at Oremeji area of Ibadan Oyo state Nigeria and the SCB was obtained from a sugarcane juice factory dumpsite in Ajah, Lagos state, Nigeria. The feedstocks were sundried immediately after collection and the dried samples were stored in air-tight plastic bags.

### 2.2 Pre-treatment of corncob and sugarcane bagasse

The sundried CC and SCB were milled using a locally fabricated grinding machine to particle size of about 0.5–2.0 mm for CC and 0.125–2.0 mm for SCB.

Pre-treatment of the substrates was performed using MWA and LHW pre-treatment methods. The MWA pre-treatment was carried out according to the method described by 26,using a microwave oven [Ethos Up, High Performance Microwave Digestion System]. Substrates were loaded into 10 mL vials at a loading of 0.2 g of substrate in 10 mL of solvent, using 0.4 M acetic acid as solvent, and treated at 120°C for 20 mins. The liquid hot water pre-treatment was performed according to the method of 27,using an oil bath [JULABO Circulator]. Samples were loaded into 150 mL stainless steel reactors, with a solid loading of 10% w/v (10 g substrate in 100 mL Milli-Q water). The reactors were then placed in an oil bath set to 160°C, for 10 min. Thereafter, the pre-treated samples were removed from the reactor as the solid fraction was separated from the liquid fraction. The solid fraction was then dried in an oven at 70°C overnight. Finally, the dried samples were stored in air tight plastic containers at room temperature prior to enzymatic hydrolysis.

### 2.3 Enzymatic hydrolysis of corncob and sugarcane bagasse

The microwave or liquid hot water pre-treated CC and SCB samples were enzymatically hydrolyzed by using a method described by Nair *et al*. [28]. Cellulase enzyme, Cellic Ctec2 (Novozymes, Denmark), with an enzyme activity of 130 FPU/mL was applied, and the substrate loading used was 7.5% w/v, with an enzyme load of 15 FPU/g dry weight of substrate. The hydrolysis was performed using 250 mL Erlenmeyer flasks with working volume of 200 mL at pH 5.5 ± 0.2, and at 35°C with 125 rpm in a shaking water bath (Grant OLS 200, Grant instrument Ltd, UK). Samples were taken regularly, at 0, 4, 8, 12, 36 and 48 hours. The amounts of sugars released were quantified using high-performance liquid chromatography (HPLC).

### 2.4 Isolation and identification of cellulose producing bacteria

Cellulose producing bacteria was isolated either from rotten banana gotten from Oje market in Ibadan, Nigeria or from Kombucha drink (Roots of Malmö Kombucha), in Sweden. Approximately 1 g of the banana sample was incubated in 4% ethanol for 7 days [29], and furthermore, 1 mL of the kombucha drink was incubated in sterile saline at 30°C with shaking at 100 rpm in a water bath shaker (Grant OLS 200, Grant instrument Ltd, UK) for 18–24 hours. The samples were serially diluted with sterile water and spread onto GYC agar containing (g L^−1^) glucose, 3; yeast [30]extract,10; CaCO_3_, 10; agar, 15. Colonies that produced clear zone of solubilization of CaCO_3_ were selected and purified. The colonies were purified by repeated streaking on Hestrin and Schramm (HS) Agar (2% glucose, 0.5% yeast extract, 0.5% peptone, 0.115% citric acid, 0.27% Na_2_HPO_4_, 1.5% agar) at pH 6.0, and incubated at 30°C for 3 days [31]. The purified cultures were grown in HS broth and the production of pellicle at the air-liquid interface of the medium was then followed up. The selected strains were stored in 30% glycerol broth at −20°C prior to further use.

Identification of the selected cellulose-producing strains was done using 16S rRNA gene sequence analysis at the Culture Collection of the University of Gothenburg, Sweden. The DNA of the isolates were extracted, and amplified using Polymerase chain reaction (PCR), thereafter, the gene were sequenced, using the method of 32. The sequences were compared to known sequences in the Genebank. The two strains selected for further investigations were identified as *Komagataeibacter* sp. CCUG73629 (Accension number OM779139) and *Komagataeibacter* sp. CCUG73630 (Accension numbers OM779138)

### 2.5 Production of biocellulose using corncob and sugarcane bagasse hydrolyzates

Precultures of *Komagataeibacter* sp. CCUG73629 and *Komagataeibacter* sp. CCUG73630 were performed in flasks containing HS medium and incubated statically at 30°C for 2–3 days. The pH was adjusted to 6.0 using 5 M NaOH or 5 M HCl prior to incubation. The enzymatic hydrolyzates of CC and SCB supplemented with other nutrients (Table 1) were then used as substrates for the BC production applying static conditions, continuous agitation, or intermittent agitation. Seed cultures, prepared as described above, were transferred into the CC or SCB enzymatic hydrolyzates (Table 1), achieving 5 mL of seed culture in 100 mL of hydrolyzate for each assay, and then the fermentation was performed in blue-capped bottles that were loosely capped under the three different conditions at 30°C for 10 days, as it is shown in Table 1. Samples were taken regularly from all assays to monitor the substrate consumption pattern of the strains during BC production. All assays were performed in duplicates.

**Table 1.** The composition of different fermentation media for bacterial cellulose production under different conditions

Production Conditions	Carbon source	Yeast Extract	Peptone	Citric acid	Na_2_HPO_4_	pH
Static	CC/SCB enzymatic hydrolyzate supplemented	5.0 g/L	5.0 g/L	1.1 g/L	2.7 g/L	6.0
Continuous Agitation (100 rpm)	CC/SCB enzymatic hydrolyzate Supplemented	5.0 g/L	5.0 g/L	1.1 g/L	2.7 g/L	6.0
Intermittent Agitation (100 rpm for 6 hours daily)	CC/SCB enzymatic hydrolyzate supplemented	5.0 g/L	5.0 g/L	1.1 g/L	2.7 g/L	6.0
Static	CC/SCB enzymatic hydrolyzate (unsupplemented)	-	-	-	-	6.0
HS medium (Static)	Glucose	5.0 g/L	5.0 g/L	1.1 g/L	2.7 g/L	6.0

### 2.6 Treatment and purification of bacterial cellulose

After fermentation, BC pellicles were harvested and purified using 1 M NaOH at 80°C for 1 hour, to remove all remnant cells and medium components and then washed with distilled water until pH 7 was reached. Finally, the BC pellicles were air dried overnight and then kept in air-tight plastic bags until further investigations.

### 2.7 Analytical methods

Compositional analysis of the untreated and pre-treated CC and SCB was done using the NREL method to determine the total solids, structural carbohydrate, and lignin components [33]. The concentration of the various components in the hydrolyzate and/or fermentation medium was analyzed by HPLC (Walters Corporation, Milford, USA). A hydrogen-based column (Aminex HPX-87 H, Bio-Rad, Hercules, USA) working at 60°C using 0.6 mL/min of 5 mM H_2_SO_4_ solution as the eluent, was used for the detection and quantification of sugars, acetic acid, and glycerol.

At the end of the fermentation, the final pH of the broth was recorded using a pH meter. The dry weight of the BC pellicle after drying was determined and expressed as gram dry weight of cellulose per liter of fermenting medium (g/L). BC yield and moisture content were determined as follows;

% BC Yield = $\frac{BC\;\;dry\;\;weight}{Carbon\;\;source\;\;used}\times 100$

BC dry weight (g/L) = the weight of BC after drying

Carbon Source used (g/L) = the amount of the carbon source used for BC production in g/L.

% Moisture content = $\frac{wet\;\;weight-dry\;\;weight}{wet\;\;weight}\times 100$

### 2.8 Statistical analysis

Statistical analysis of the results obtained were performed using MINITAB 17.0 Software. Analysis of variance (ANOVA) was performed using general linear models with 95% confidence interval, followed by Tukey’s pairwise comparison test. All experiments were performed in duplicate and error bars presented on the graphs represent two standard deviations.

### 2.9 Characterization of bacterial cellulose.

#### 2.9.1 Fourier Transformed Infra-red spectroscopy (FTIR)

FTIR was performed using a FTIR spectrometer (Nicolet iS10, Thermo Fisher Scientific, Waltham, USA). The BC samples were analyzed by placing the dried film on the diamond accessory. The FTIR spectra were recorded in the range of 4000–500 cm^−1^ wavenumbers, with an accumulation of 32 scans.

#### 2.9.2 Field-Emission Scanning Electron Microscopy (FE-SEM)

Surface morphology of the BC film was studied by FE-SEM (Zeiss, Sigma, Germany) imaging. The films were attached to a carbon tape and covered with gold. Photomicrographs were taken at 8,000, 15,000, and 20,000 x magnifications, using an accelerating voltage of 5 kV.

#### 2.9.3 Thermogravimetric Analysis (TGA)

Thermogravimetric analysis of dried BC films was performed on a TA instrument (Q500 TA instrument, Waters LLC, New Castle, DE, USA), to determine the thermal properties of the BC. The samples, with a weight of 5–10 mg, were heated in aluminia pans from room temperature to 600°C at a heating rate of 20°C/min in a nitrogen atmosphere with a flow rate of 10.0 mL/min.

#### 2.9.4 Mechanical Properties

The mechanical properties of BC were determined by a dynamic mechanical analyzer (DMA) (DMA Q800, TA instruments, Waters LLC, USA). The analysis was operated in a stress/strain mode, using a tension film clamp. The film was cut in a typical width of 5.3 mm, with a length of approximately 17 mm. The test was performed at room temperature. The stress (σ), strain (ℇ and Young’s modulus were then calculated.

## 3 Results and discussion

Generally, the production of BC has a direct impact on its supramolecular structure, mechanical and physical properties [15], there is therefore a need to investigate cost-effective and sustainable technologies, substrates, culture conditions as well as different strains for BC production. This study investigated BC production, using CC and SCB as substrates. Both CC and SBC are widely available cheap lignocellulose-rich substrates, however, due to the recalcitrant structure of lignocelluloses, these types of substrates need to be subjected to pretreatment aiming to liberate fermentable sugars. Environmentally friendly LHW, and MWA pretreatment methods were applied to break up the lignocellulose structure prior to enzymatic hydrolysis. The obtained enzymatic hydrolyzates were then subjected to fermentation using two different *Komagataeibacter* sp. under different conditions, i.e., static, as well as using continuous and intermitten agitation. Finally, physicochemical properties of the produced BC were determined using methods as FTIR, FE-SEM, TGA, and DMA.

### 3.1 Pre-treatment and enzymatic hydrolysis of the substrates

Pre-treatment of CC and SCB, using MWA and LHW pre-treatment methods, was investigated, in order to determine the best method for enhanced enzymatic hydrolysis.

Pre-treatment of lignocellulosic biomass is generally applied aiming to improve enzymatic digestibility of the substrate [26]. Enzymatic hydrolysis of both untreated and pre-treated CC and SCB resulted in higher glucose concentrations than that of pentoses (Table 2), due to a higher cellulose content compared to that of hemicelluloses, as it was also determined by the compositional analysis of the untreated substrates. Furthermore, the pre-treatment applied resulted in a somewhat higher released sugars after the enzymatic hydrolysis in case of CC, however the difference obtained for untreated *vs* treated CC was not statistically significant (Table 2). Since according to the results, there was no difference obtained regarding the effectiveness of MWA or LHW, LHW pre-treatement was chosen for the subsequent investigations, as it is highly efficient and economically feasible, as well as environmentally friendly, reducing the usage of chemicals and their effects on the environment. The LHW dissolves hemicelluloses and lignin, which are transferred into the liquid phase, while leaving cellulose as solid. Consequently, the biomass digestibility increases as cellulose becomes more accessible to enzymatic hydrolysis, where partial hydrolysis of cellulose can occur as a result of acetic acid formation [34].

**Table 2.** Sugar concentrations obtained in enzymatic hydrolyzates of untreated and pre-treated corncob (CC) and sugarcane bagasse (SCB) after 48 hrs of enzymatic hydrolysis

	CC			SCB	
	Untreated (g/L)	Microwave Assisted (g/L)	Liquid Hot water (g/L)	Untreated (g/L)	Liquid HotWater (g/L)
Hexose	14.5 ± 0.5^a^	16.3^a^	15.9 ± 0.8^a^	20.6 ± 1.2^a^	15.8 ± 1.9^a^
Pentose	7.8 ± 0.1^b^	10.8^a^	10.1 ± 0.4^a^	12.6 ± 1.1^a^	9.6 ± 1.0^a^

Each values represent mean of replicate, alphabets as superscript across rows indicates a significant difference p ≤ 0.05 by Tukey test

Enzymatic hydrolysis of SCB showed that the glucose and pentose yield was higher (20.6 g/L and 12.6 g/L respectively) in the hydrolyzate of untreated (milled) SCB than those in the hydrolyzate of LHW pre-treated SCB, which had glucose and pentose yield of 15.8 g/L and 9.6 g/L, respectively (Table 2). This is contrary to the report of 26, who recorded higher C5 and glucose yields after using MWA oxalic acid pre-treatment and then hydrolysis. However, in this study a milled, that is, mechanically pre-treated SCB was subjected to further pre-treatment with only LHW, as the samples were lost during MWA pre-treatment experimentation due to technical difficulties. Milling aiming to reduce the particle size, will lead to an increase in accessible surface area, which in turn can improve enzymatic digestibility [26]. Moreover, milling as a mechanical size reduction provides a non-chemical, green route for pre-treatment of lignocellulosic materials [22].

### 3.2 Bacterial cellulose production using lignocellulosic biomass as substrate

When lignocellulosic biomass is subjected to a pre-treatment one of the goals is to make cellulose accessible for the subsequent hydrolysis steps to release sugars. It may rise the question, why do one need to convert cellulose present in lignocellulosic biomass to cellulose produced by bacteria. The production of bacterial cellulose has the benefits of purer cellulose without lignin and hemicellulose, easy to extract, nontoxic, biodegradable with human compatibility. After enzymatic hydrolysis of a substrate, the hydrolyzate may contain compounds that can potentially stimulate the synthesis of a polysaccharide or substances, which may act inhibitory to cell growth and metabolism [35]. Bacteria of different genera have the ability to utilize different sugars for growth and metabolism. *Komagataeibacter* sp. are acetic acid bacteria known to produce cellulose in large quantities, as they also have the ability to utilize a wide variety of substrates [8].

In this study, BC production from CC and SCB hydrolyzate was investigated using two different strains of *Komagataeibacter* sp., *Komagataeibacter* sp. CCUG73629 and *Komagataeibacter* sp. CCUG73630. Both strains showed the ability to consume glucose and pentose sugars during fermentation leading to the production of bacterial cellulose.

Generally, during cellulose synthesis, glucose acts as an energy source as well as a precursor for the synthesis [36]. Media supplementation with nitrogen and phosphate sources improved BC production and yield. Moreover, it was found that the use of agitation will also enhance BC production, because better air diffusion into the media and then to the cells will increase metabolic activity and thereby the production rate of BC [37]. Hence, aerobic cells, like acetic acid bacteria, will access higher oxygen circulation, which may enhance their metabolic activity. Furthermore, there was a reduction in the pH observed after BC production by both strains in the production media (Table 3–6), which may be the result of the accumulation of acids produced by the strains. These strains are acetic acid bacteria, with the ability to synthesize acetic acid, and moreover these strains have also the ability to utilize the acetic acid that has been synthesized [38].

**Table 3.** Bacterial cellulose production by *komagataeibacter* sp. CCUG73629 from corncob (CC) at different fermentation conditions

Production Media	BC yield (%)	Final pH	Moisture content (%)
Static (supplemented)	8.6 ± 1.7^ab^	4.6 ± 0.1^a^	99.3 ± 0.0^a^
C.Agitation (Supplemented)	14.1 ± 2.7^a^	4.5 ± 0.1^a^	99.2 ± 0.1^a^
I.Agitation (Supplemented)	11.9 ± 0.0^a^	4.5 ± 0.0^a^	99.3 ± 0.1^a^
Static (unsupplemented)	2.6 ± 0.3^b^	3.2 ± 0.1^c^	98.9 ± 0.1^b^
HSM	7.9 ± 1.5^ab^	3.6 ± 0.0^b^	99.5 ± 0.1^a^

Each values represent mean of replicate, any letter found as superscript along columns indicates a significant difference p ≤ 0.05 by Tukey test

**Table 4.** Bacterial cellulose production by *komagataeibacter* sp. CCUG73630 from corncob (CC) at different fermentation conditions

Production Media	BC yield (%)	Final pH	Moisture content (%)
Static (supplemented)	3.0 ± 1.0^ab^	4.3 ± 0.0^b^	99.8 ± 0.0^a^
C.Agitation (Supplemented)	3.0 ± 0.4^ab^	4.3 ± 0.0^b^	99.7 ± 0.0^a^
I.Agitation (Supplemented)	3.4 ± 1.6^ab^	4.2 ± 0.1^b^	99.8 ± 0.1^a^
Static (unsupplemented)	-	5.4 ± 0.0^a^	-
HSM	4.5 ± 0.7^a^	3.5 ± 0.1^c^	99.7 ± 0.1^a^

Each values represent mean of replicate, any letter found as superscript along columns indicates a significant difference p ≤ 0.05 by Tukey test

**Table 5.** Bacterial cellulose production by *komagataeibacter* sp. CCUG73629 from sugarcane bagasse (SCB) at different fermentation conditions

Production Media	BC yield (%)	Final pH	Moisture content (%)
Static (supplemented)	5.9 ± 1.9^ab^	4.5 ± 0.1^a^	99.3 ± 0.2^a^
C.Agitation (Supplemented)	9.4 ± 1.4^a^	4.7 ± 0.0^a^	99.3 ± 0.1^a^
I.Agitation (Supplemented)	7.9 ± 1.9^ab^	4.5 ± 0.1^a^	99.3 ± 0.0^a^
Static (unsupplemented)	1.7 ± 1.0^b^	3.3 ± 0.0^c^	99.4 ± 0.1^a^
HSM	3.2 ± 2.4^ab^	4.1 ± 0.0^b^	99.5 ± 0.0^a^

Each values represent mean of replicate, any letter found as superscript along columns indicates a significant difference p ≤ 0.05 by Tukey test

**Table 6.** Bacterial cellulose production by *Komagataeibacter* sp. CCUG73630 from sugarcane bagasse (SCB) at different fermentation conditions

Production Media	BC yield (%)	Final pH	Moisture content (%)
Static (supplemented)	6.2 ± 0.2^b^	4.4 ± 0.1^a^	99.9 ± 0.0^a^
C.Agitation (Supplemented)	12.7 ± 1.5^a^	4.3 ± 0.0^a^	99.9 ± 0.0^a^
I.Agitation (Supplemented)	4.7 ± 0.1^b^	4.3 ± 0.0^a^	99.9 ± 0.0^a^
Static (unsupplemented)	1.5 ± 0.3^b^	3.9 ± 0.8^a^	99.8 ± 0.2^a^
HSM	4.4 ± 2.7^b^	3.7 ± 0.1^a^	99.9 ± 0.0^a^

Each values represent mean of replicate, any letter found as superscript along columns indicates a significant difference p ≤ 0.05 by Tukey test

**Figure 1.**
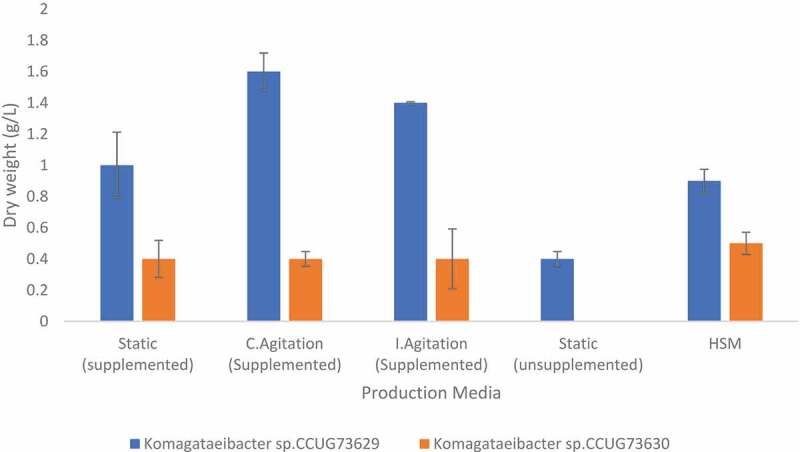
Dry weight of BC produced by *Komagataeibacter* sp. CCUG73629 and *Komagataeibacter* sp. CCUG73630 in corncob (CC) hydrolyzate at different fermentation conditions.

During BC production, the highest BC yields, i.e., 14.1 and 11.9% could be achieved at respectively, continuous and intermittent agitation conditions by *Komagataeibacter* sp. CCUG73629 in CC hydrolyzate (Table 3). While defined HS medium gave the highest BC yield by *Komagataeibacter* sp. CCUG73630, nevertheless it was much lower, i.e., 4.5% (Table 4) than those observed in any conditions in CC hydrolyzate, whereas *Komagataeibacter* sp. CCUG73629 gave a yield of 7.9% in defined HS medium (Table 3). This means that, the complex composition of CC hydrolyzate clearly favored the BC production process by *Komagataeibacter* sp. CCUG73629. The produced dry weight of BC obtained during continuous and intermittent agitation *i.e*. 1.6 g/L and 1.4 g/L, respectively (Figure 1), were higher than that of 0.65 g/L observed during agitation in CC acid hydrolyzate reported by 24.

Although, the use of HS medium supported a higher yield of BC produced by *Komagataeibacter* sp. CCUG73630, because the strain thrives best in glucose predominant medium, which was also confirmed here in this study. Previous reports on the production of BC using wastes and by-products shows that the utilization of some of the waste materials resulted in higher BC yield than those obtained with the use of HS medium. For instance, waste from lipid fermentation resulted in a BC production of 0.4–0.6 g/L [39] while from molasses the production of BC was 1.64 g/L [40], and using olive oil mill wastewater as substrate gave a higher BC of 5.33 g/L than that observed in HS medium [14]. However, 41,reported a higher BC production in HS (6.7 g/L) than that from cashew tree exudate and cashew gum (2.8 g/L and 2.3 g/L, respectively). 42,also reported that BC produced by *Gluconacetobacter xylinum* from polysaccharide wastewater was lower (1.177 g/L) than that produced by the same strain in HS medium (1.757 g/L).

There was no significant difference between the BC yields observed under static *vs* agitation conditions in cases when *Komagataeibacter* sp. CCUG73630 was cultivated in CC hydrolyzate, meaning that different cultivation conditions did not have any significant effect on the BC yield (Table 4). This is in line with 43, who found that there was no statistically significant difference in the yield of cellulose produced by *Gluconacetobacter xylinus* in the same media under static and agitated condition. They explained that although oxygen might be a limiting factor in cellulose production, however, allowing access to more oxygen by agitating did not increase the cellulose yields.

**Figure 2.**
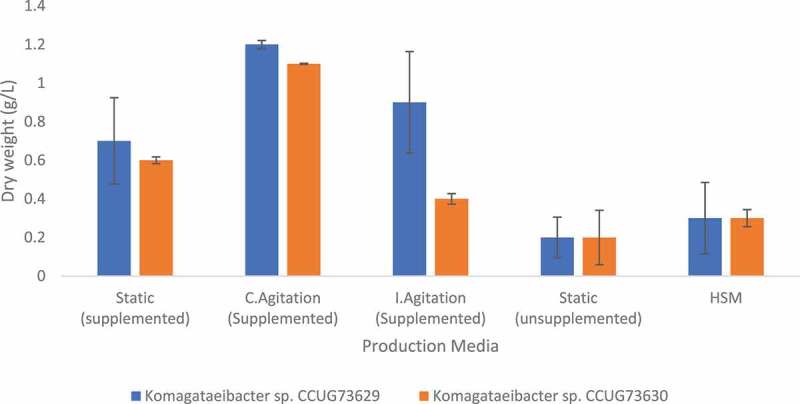
Dry weight of BC produced by *Komagataeibacter* sp. CCUG73629 and *Komagataeibacter* sp. CCUG73630 in sugarcane bagasse (SBC) hydrolyzate at different fermentation conditions.

In SCB hydrolyzate, continuous agitation increased the BC yield in case of both strains, i.e., 9.4% and 12.7% were observed with *Komagataeibacter* sp. CCUG73629 (Table 5) and *Komagataeibacter* sp. CCUG73630 (Table 6), respectively. While intermittent agitation resulted in a higher BC yield (7.9%) for *Komagataeibacter* sp. CCUG73629 (Table 5) than that (4.7%) with *Komagataeibacter* sp. CCUG73630 (Table 6). The production of BC was higher, i.e., 1.2 g/L dry weight, under continuous agitation in SCB hydrolyzate (Figure 2) than that recorded by 38,who reported a production of 1.09 g/L and 0.42 g/L in bagasse acid and enzymatic hydrolyzate, respectively.

Agitation generally enhanced BC yield greatly compared to that observed under static culture condition. In static condition, as BC mass increases in the medium, oxygen circulation reduces, with cells having little or no access to oxygen. Oxygen has been reported to be a limiting factor in bacterial cellulose production [44], thus allowing access to oxygen via agitation increases even access to nutrients and thereby increases metabolic activity [45]. Aydin and Aksoy [46] reported 120 rpm as the best agitation condition for cell growth and BC production by *Gluconacetobacter hansenii* P2A, while 37,reported agitation at 200 rpm for BC production by *Gluconacetobacter hansenii* NCIM 2529. 47,reported 150 rpm as the maximum agitation speed for cellulose production by *Acetobacter xylinum* KJ-1.

The low yield of BC in the CC hydrolyzate not supplemented with nitrogen and phosphate sources, i.e., 2.6% and zero production, by *Komagataeibacter* sp. CCUG73629 (Table 3) and *Komagataeibacter* sp. CCUG73630 (Table 4), respectively, emphasizes the importance of nutritional supplements in a culture medium to favor BC production [48]. Also, a lower yield of BC *i.e*. 1.7% and 1.2% by *Komagataeibacter* sp. CCUG73629 (Table 5) and *Komagataeibacter* sp. CCUG73630 (Table 6), respectively, was recorded in the unsupplemented SCB hydrolyzate, when compared with those produced in SCB hydrolyzate supplemented with nitrogen and phosphate sources. Yeast extract and peptone as organic nitrogen source, when added to culture medium for BC production is indispensable for a significant BC production [13,49]. This also agrees with Coban and Biyik [50], who reported a higher BC production in a glucose media supplemented with yeast extract as nitrogen source.

### 3.3 Characterization of bacterial cellulose

After production of a biomaterial, it is important to investigate the structural features because the physicochemical properties of such materials can be influenced by the composition and physical properties of the culture medium. In this study, it was observed that the fermentation condition and type of substrate had impact on some of the structural and physicochemical properties.

#### 3.3.1 Chemical structure of bacterial cellulose

**Figure 3.**
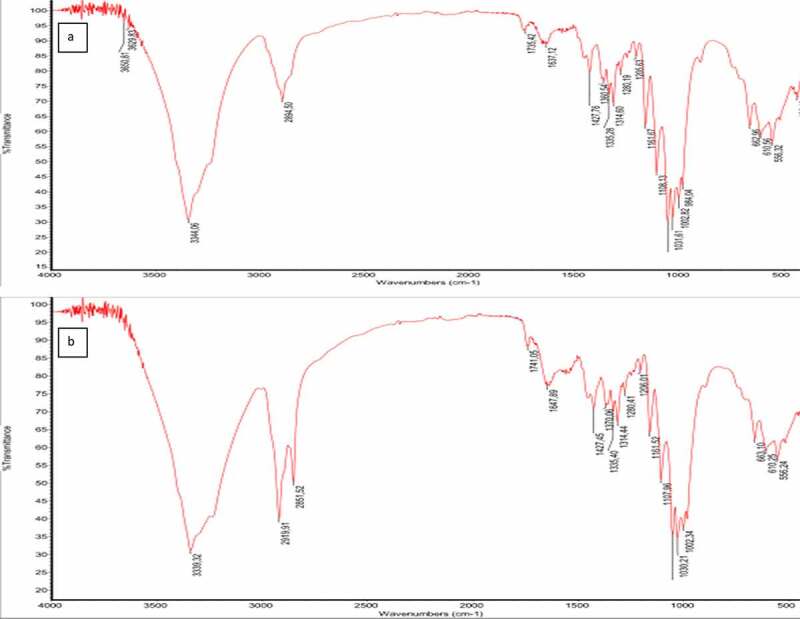
FTIR spectra of BC produced by *Komagataeibacter* sp. CCUG73629 from sugarcane bagasse (SCB) hydrolyzate at different fermentation conditions.

The spectra of all BC produced under different conditions were identical and exhibited characteristic bands of cellulose I. The hydroxyl, aldehyde, alkane, and alkene functional groups were present in all samples. The functional groups and fingerprint regions associated with cellulose can be found between 1800 and 500 cm^−1^; that is, peaks around 1647 cm^−1^ indicating CO stretching, peaks around 1427 cm^−1^ indicating – OH bending, peaks around 1160 cm^−1^ indicating C-O-C asymmetric stretching at β – glycosidic linkage, peaks around 1108 cm^−1^ indicating C-O bond stretching, peaks around 1030 cm^−1^ indicating C-O-C ring skeletal vibration, and peaks around 1314 cm^−1^ indicating CH_2_ wagging at C-6 were associated with cellulose. It was observed that only BC produced under agitation in SCB by *Komagataeibacter* sp. CCUG73629 (Figure 3), had slightly different spectra, presenting two peaks at around 2919 and 2851 cm^−1^representing C-H stretching vibration of sugar rings. These probably occur due to the agitation as only single peaks were observed for BC obtained at static conditions. Similar functional groups and peaks for BC have been reported by various researchers [51–53].

The absence of peaks around 1540 cm^−1^ and 1640 cm^−1^ which corresponds to amine bonds, are associated with proteins from culture media or residual bacterial biomass. Their absence indicates that the BC membrane was properly cleaned and is pure [54,54].

The absence of peaks around 3440 cm^−1^ to 3495 cm^−1^ indicating OH stretching due to intramolecular hydrogen bonds confirms the absence of cellulose II [55,56]. This therefore confirms that the BC produced by *Komagataeibacter* sp. CCUG73629 and *Komagataeibacter* sp. CCUG73630 under different fermentation conditions is cellulose I.

#### 3.3.2 Morphology of bacterial cellulose

The morphology of BC determined using SEM considers the fibril density, size, and arrangement, which can be dependent on the media composition, viscosity, and activity of the BC producing bacteria [45,57].

**Figure 4.**
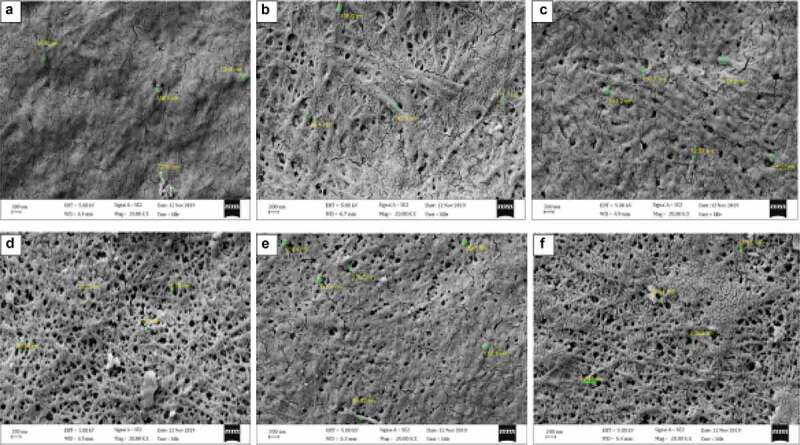
Scanning electron micrograph of BC produced from corncob (CC) hydrolyzate by *Komagataeibacter sp* CCUG73629 (a-c) and *Komagataeibacter sp* CCUG73630 (d-f) under (a, d) Static, (b, e) Intermittent agitation, and (c, f) Continuous agitation conditions at a magnification of 20,000 X.

**Figure 5.**
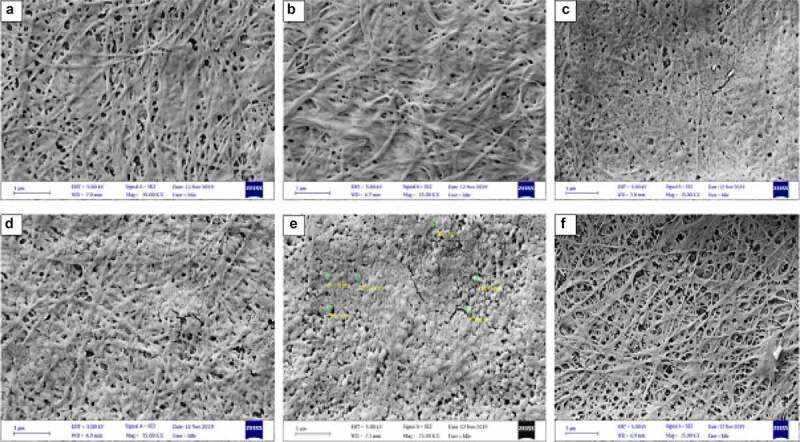
Scanning electron micrograph of BC produced from sugarcane (SCB) hydrolyzate by *Komagataeibacter* sp. CCUG73629 (a-c) and *Komagataeibacter* sp. CCUG73630 (d-f) under (a, d) Static, (b, e) Intermittent agitation and (c, f) Continuous agitation conditions at a magnification of 15,000 X.

BC produced by *Komagataeibacter* sp. CCUG73629 and *Komagataeibacter* sp. CCUG73630 in SCB and CC hydrolyzate medium were densely packed showing thin BC fibers (Fig 4 and 5). Although, most of the BC fibers displayed longer fibrous networks, they had varying microfibril diameters ranging from 42 to 120 nm. The densely packed network of cellulose with thinner fibers indicate that BC has more hydrogen-bonding pattern, a more compact pattern that may result in higher tensile strength of the BC [58].

10,stated that no major variations were observed in the dimension of the four bacterial nano cellulose produced. However, in the present study, variations were observed in the BC morphology, which we believe are due to the different fermentation conditions, bacterial strains, and the production medium applied. For example, a porous network with thin fibers were observed in BC produced by *Komagataeibacter* sp. CCUG73630 under static condition in CC hydrolyzate (Figure 4d) than BC produced by *Komagataeibacter* sp. CCUG73629 under static condition (Figure 4a) in the same medium. The porous nature within the fibril arrangement gives BC high porosity and water accumulating properties, which are responsible for water retention, an important property for application in biomedicine [45].

#### 3.4.3 Thermal properties

The thermal properties of BC is determined by its thermal stability during degradation. Cellulose degradation shows loss of weight due to degradation and decomposition of the glycosyl units. The BC produced by *Komagataeibacter* sp. CCUG73629 and *Komagataeibacter* sp. CCUG73630 in CC and SCB hydrolyzate medium under all fermentation conditions showed higher stability, *i.e*. (396–364°C) and (314–399°C), respectively, except those produced by *Komagataeibacter* sp. CCUG73630 under continuous agitation in SCB (314°C) (Table 7). Furthermore, the BC produced in the CC and SCB hydrolyzate by *Komagataeibacter* sp. CCUG73629 and *Komagataeibacter sp*. CCUG73630 showed higher thermal stability than those produced in the defined HS medium *i.e*. 353°C and 346°C, respectively (Table 7). BC-producing medium can form a more effective chemical interactions (like hydrogen bonds) with hydroxyls group of bacterial cellulose and hence increase its thermal stability [41]. Thermal degradation of cellulose skeleton starts around 220°C and overall degradation is completed above 300°C; thus, the variations in the degradation temperature may be due to the variations in fibril size, arrangement, and compactness, with higher thermal degradation indicating higher crystallinity [45]. Maximum degradation temperature is a criterion for thermal stability.

**Table 7.** Maximum degradation temperature of bacterial cellulose produced by *Komagataeibacter* sp. CCUG73629 and *Komagataeibacter* sp. CCUG73630 from corncob (CC) and sugarcane bagasse (SCB) hydrolyzate

	*Komagataeibacter* sp. CCUG73629		*Komagataeibacter* sp. CCUG73630	
Production media/Condition	CC	SCB	CC	SCB
	Maximum degradation temperature (°C)		Maximum degradation temperature (°C)	
Static (supplemented)	387.2	372.8	370.6	399.3, 445.8
C.Agitation (Supplemented)	372.2	364.8	384.5	314.5
I.Agitation (Supplemented)	346.0	396.5	374.6	342.4
Static (unsupplemented)	389.5	395.3	-	395.9
HSM	353.3		346.5	

#### 3.4.4 Mechanical properties

**Figure 6.**
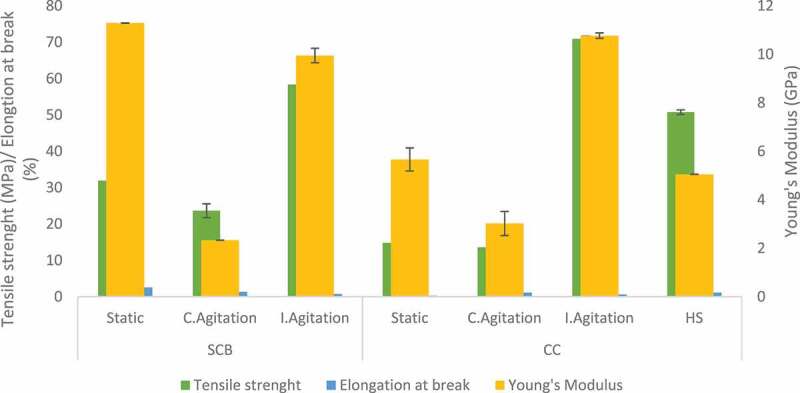
Tensile strength, Elongation at break and Young’s modulus of BC produced by *Komagataeibacter* sp. CCUG73630 from sugarcane bagasse (SCB) and corncob (CC) hydrolyzate at different fermentation conditions.

**Figure 7.**
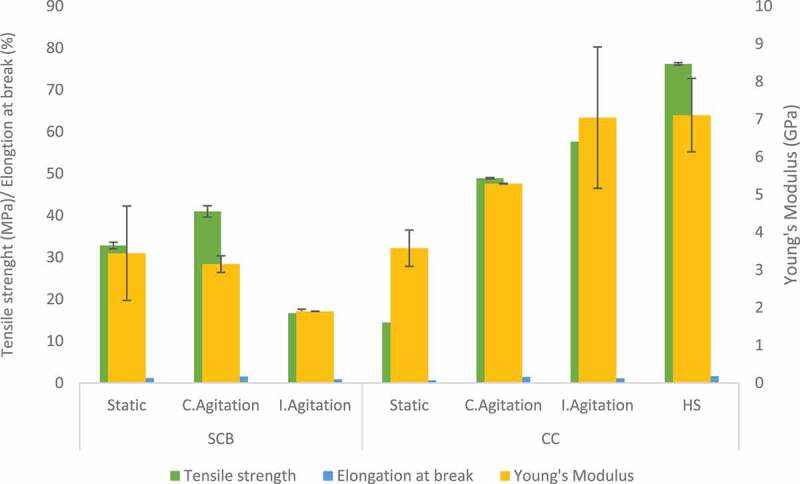
Tensile strength, Elongation at break and Young’s modulus of BC produced by *Komagataeibacter* sp. CCUG73629 from sugarcane bagasse (SCB) and corncob (CC) hydrolyzate at different fermentation conditions.

The mechanical properties of BC are dependent on the physical nature of the fibrils together with the strength of intermolecular hydrogen bonding between the cellulose chains [59]. Moreover, the fermentation condition can also influence the mechanical properties of BC. DMA was used for these investigations in this study due to the size of the BC film obtained. BC produced by *Komagataeibacter hansenii* CCUG73629 in HS medium had a high modulus of elasticity (7.1 GPa) and tensile strength (76.2 MPa) (Figure 7). Intermittent agitation increased the mechanical properties of BC produced by both strains in CC hydrolyzate medium (Fig. 6 and 7) and *Komagataeibacter* sp. CCUG73630 in SCB hydrolyzate medium (Figure 6). BC produced under intermittent agitation by *Komagataeibacter* sp. CCUG73629 in CC had modulus of 7.0 GPa and tensile strength of 57.8 MPa (Figure 7). In addition, BC produced by *Komagataeibacter* sp. CCUG73630 under intermittent agitation in CC had modulus of 10.8 GPa and tensile strength of 70.9 MPa, while in SCB, had modulus of 9.9 GPa and tensile strength of 58.4 MPa (Figure 6). The high modulus of elasticity of BC produced by both strains under intermittent agitation showed that the intermittent agitation supported production of a stiffer BC, with strong hydrogen bonding.

42,reported that BC produced by *G. xylinum* in HS medium had a higher modulus of elasticity, tensile strength, and strain at break when compared to BC produced in polysaccharide fermentation wastewater. 60,also reported that BC produced in glucose, yeast extract, and peptone medium by *G. hansenii* had tensile strength of 76.7 MPa. 56,also reported tensile strength of 46.9 MPa and a modulus of 3.2 GPa for BC produced by *Komagataeibacter rhaeticus*.

The mechanical properties depicts high strength of BC samples, as the fibril size and arrangement also influences these properties. Uniform size of well-arranged fibrils provides higher strength to BC [45]. The modulus of elasticity of a polymer relates directly to the stiffness of the material, i.e., the higher the modulus of elasticity, the stiffer the material [61].

## Conclusion

Bacterial cellulose, an extracellular matrix secreted by bacteria is an appropriate alternative to plant cellulose, with excellent physicochemical properties that makes it suitable for several applications. This research showed that CC and SCB after applying mechanical and LHW pretreatments prior to enzymatic hydrolysis are suitable substrates for BC production. Intermittent agitation proved to be a promising alternative to continuous agitation with good BC yield and properties. Furthermore, it was also found that different fermentation conditions can influence the physicochemical properties of the BC.
